# Extracellular RNA as a molecular driver and therapeutic target in abdominal aortic aneurysms

**DOI:** 10.1038/s41598-025-22041-y

**Published:** 2025-10-31

**Authors:** Nahla Ibrahim, Hubert Hayden, Gabriel Kurzreiter, Johannes Klopf, Sonja Bleichert, Tyler Artner, Alexander Stiglbauer-Tscholakoff, Wolf Eilenberg, Christoph Neumayer, Christine Brostjan

**Affiliations:** 1 Division of Vascular Surgery, Department of General Surgery, Medical University of Vienna and University Hospital Vienna, Vienna, Austria; 2 Division of Cardiology, Department of Internal Medicine II, Medical University of Vienna and University Hospital Vienna, Vienna, Austria; 3 Division of Cardiovascular and Interventional Radiology, Division of Molecular and Gender Imaging, Department of Biomedical Imaging and Image Guided Therapy, Medical University of Vienna and University Hospital Vienna, Vienna, Austria

**Keywords:** Abdominal aortic aneurysm, Extracellular RNA, Extracellular DNA, Mouse models, Cell free RNA, Cell free DNA, Diseases, Medical research, Molecular medicine, Pathogenesis

## Abstract

Abdominal aortic aneurysms (AAAs) are characterized by chronic inflammation, matrix degradation and smooth muscle cell (SMC) loss, leading to vessel dilation and rupture, with no current pharmaceutical management options. Since recent studies have highlighted the role of extracellular (ex) nucleic acids in promoting inflammation and tissue damage in cardiovascular conditions, we aimed to characterize the contribution of exDNA and exRNA to AAA pathogenesis and evaluate their potential as therapeutic targets in established disease. Circulating exDNA was elevated in patients and mouse models, while plasma levels of exRNA were not associated with AAA development. When RNase A or DNase I was administered to mice with established disease, the targeted degradation of exRNA, but not exDNA, significantly attenuated aneurysm growth. The RNase A treatment produced systemic anti-inflammatory effects (reduced monocyte/granulocyte count) and showed the potential to improve local vascular conditions by preserving SMC integrity, reducing macrophage infiltration and protease expression. These effects were not observed in DNase I-treated animals. In conclusion, while circulating exDNA showed AAA biomarker potential, targeting exRNA by systemic RNase A treatment effectively mitigated aneurysm progression in established disease through pleiotropic modulation of inflammation and tissue remodeling, presenting a novel and promising therapeutic strategy.

**Supplementary Information:**

The online version contains supplementary material available at 10.1038/s41598-025-22041-y.

## Introduction

Abdominal aortic aneurysm (AAA) is a life-threatening condition characterized by progressive dilation of the abdominal aorta, leading to an increased risk of aortic rupture and significant associated mortality^1,2^. The pathogenesis of AAA is complex, involving chronic inflammation, extracellular matrix degradation, immune cell infiltration, oxidative stress, and vascular smooth muscle cell (SMC) death or de-differentiation^3^. Despite extensive research, effective pharmacological therapies to halt or reverse AAA progression are still unavailable, leaving surgical intervention as the only treatment option for the disease according to current guidelines^4^.

In recent years, extracellular nucleic acids, in particular extracellular DNA (exDNA)^5^ and extracellular RNA (exRNA)^6^, have garnered attention for their potential in the propagation of vascular inflammation in various cardiovascular diseases (CVDs), including AAA^7,8^.

exDNA, comprised of DNA fragments circulating in bodily fluids like plasma, is released through various processes such as cellular apoptosis, necrosis and, particularly in the context of inflammation, from neutrophil extracellular trap (NET) formation^9,10^. Elevated levels of exDNA, presumed to be primarily of NET origin, have been associated with various CVDs and with disease severity^11–13^. In line, we have found that exDNA was significantly increased in the plasma of AAA patients in comparison to matched controls, and correlated strongly with markers of NET release^14^. In preclinical AAA models, NET formation was linked to aneurysm development^15–17^, and NET-related markers such as citrullinated histone 3 (CitH3) were found to be elevated in AAA tissue and plasma^18^. These observations have positioned exDNA and NETs as contributors to AAA pathogenesis and as potential therapeutic targets.

In parallel, extracellular RNA (exRNA) has emerged as a potent pro-inflammatory damage-associated molecular pattern (DAMP) with relevance to vascular disease^19,20^. exRNA can be released from various stressed or damaged cell types, including endothelial cells, platelets, and smooth muscle cells, and exists in both free and extracellular vesicle–associated forms^6,8^. Functionally, exRNA has been shown to trigger vascular inflammation by promoting leukocyte adhesion and cytokine expression^21^ via toll-like receptors and other pattern recognition pathways^20,22^. However, while the pro-inflammatory functions of exRNA are increasingly recognized, the extracellular actions of RNA, as signaling molecules or DAMPs, remain poorly understood in the context of AAA, and no studies to date have systematically evaluated its therapeutic targeting.

In this study, we aimed to compare exDNA and exRNA in AAA patient samples and to investigate the contribution of extracellular nucleic acids to AAA growth in two murine models. We performed side-by-side evaluation of exDNA and exRNA as therapeutic targets, and tested the effects of degrading nucleic acids by systemic DNase I or RNase A treatment. Importantly, these interventions were administered in mice with already established AAAs, providing a clinically relevant setting for evaluating treatment efficacy on AAA progression. We further assessed systemic effects on immune cell populations, as well as local changes in the aortic wall via histology and gene expression analysis.

## Materials and methods

### Ethical approval (mouse and human studies)

Animal experiments were approved by the local ethics committee and the Austrian Ministry of Science (BMWFW-66.009/0355-WF/V/3b/2016, 0248-WF/V/3b/2017, and 2020-0.547.895). All experiments were performed in accordance with the guidelines and regulations of the European Directive 2010/63/EU and the Austrian Animal Experiment Act 2012 and in accordance with the ARRIVE guidelines 2.0.

Analysis of human plasma samples from AAA patients and controls was approved by the institutional ethics committee of the Medical University of Vienna (license number 1729/2014), was conducted in accordance with The Code of Ethics of the World Medical Association (Declaration of Helsinki and current amendments) and was registered at https://www.researchregistry.com (unique identifying number 7647). Written informed consent was given by all study participants prior to blood withdrawal and their demographics were recorded by means of a structured questionnaire. The median age of participants was 63.5 years for the control cohort and 67.1 years for the AAA cohort. Other relevant demographics are summarized in Supplementary Table 1.

### Mouse models of abdominal aortic aneurysm

#### Anesthesia, analgesia, and euthanasia

All surgical procedures were performed under general anesthesia using isoflurane (1.8-2.0%) delivered in 2 L/min oxygen. Prior to operating, mice were given analgesia with buprenorphine (2.5%) administered subcutaneously at 10 µL/g body weight. Post-operatively, mice received 10% glucose (10 µL/g, subcutaneous) and were monitored during recovery under a heating lamp. To ensure sustained analgesia, animals were provided drinking water supplemented with 7.5 mg piritramide and 0.5% glucose in 200 mL for 3 days following surgery.

For euthanasia, mice were administered a terminal dose of ketamine (100 mg/kg) and xylazine (5 mg/kg) intraperitoneally.

#### Angiotensin II (AngII) AAA mouse model

AAAs were induced in male ApoE-deficient mice (strain B6.129P2-ApoEtm1Unc/J@Him; 11–15 weeks old, obtained from the Core Facility Laboratory Animal Breeding and Husbandry, Medical University of Vienna, Austria) maintained on a standard diet. Mice were implanted subcutaneously with ALZET osmotic mini-pumps (model 2004; DURECT Corporation, USA) loaded with angiotensin II (Bachem AG, Switzerland), delivering AngII continuously at 1,000 ng/kg/min for 28 days^23,24^. Baseline imaging of the suprarenal aorta was performed by high-resolution 3D ultrasound prior to pump implantation, with follow-up assessments on day 8 and day 27 post-infusion to monitor aneurysm growth^25^.

#### External Porcine pancreatic elastase (EPPE) AAA mouse model

In a second model, AAAs were induced in male C57BL/6J wild-type mice (9–12 weeks old, obtained from the Core Facility Laboratory Animal Breeding and Husbandry, Medical University of Vienna, Austria) via topical peri-adventitial application of 10 µL porcine pancreatic elastase solution (7.6 mg/mL; Sigma-Aldrich/Merck KGaA, USA) to the exposed infrarenal abdominal aorta for 5 minutes^25,26^. Imaging of the infrarenal aorta was conducted at baseline and on postoperative days 4 and 13 to assess aneurysm development^25^.

#### 3D high-resolution ultrasound imaging of AAA progression

AAA development was monitored using the Vevo 2100 or 3100 high-frequency ultrasound imaging system (FUJIFILM VisualSonics Inc., Canada). Semi-automated 3D reconstructions were obtained by acquiring 157 frames over a 12 mm segment of the abdominal aorta, as previously described^24^. Aortic volume analysis was performed using Vevo LAB software (version 5.6.1, FUJIFILM VisualSonics Inc.), and suprarenal or infrarenal aortic volume was calculated in cubic millimeters. All measurements were performed independently by two blinded observers, and average values were used for final calculations.

Aorta volume was assessed based on our previous findings that 3D volume as compared to maximum diameter measurements offer superior sensitivity for detecting progressive aneurysm growth and may reduce the number of animals required to achieve statistical power^25^.

As a procedural detail, final ultrasound measurements were conducted one day prior to tissue collection: day 27 for the AngII model and day 13 for the EPPE model. As such, ultrasound-derived AAA growth metrics refer to these respective time points, while tissue-based analyses were performed on aortas collected the following day (day 28 and day 14, respectively).

#### Intravenous drug administration and end point procedures

To evaluate therapeutic effects on established aneurysms, mice underwent treatment only if aortic volume growth exceeded 120% at the interim ultrasound time point: day 8 in the AngII model or day 4 in the EPPE model. Eligible animals were stratified into treatment or control groups by 1:1 matching based on percent aortic volume increase relative to baseline.

On the following day (day 9 for AngII, day 5 for EPPE), animals underwent catheterization of the external jugular vein for intravenous drug delivery via a single-channel vascular access button (VAB; 22 gauge, Instech Laboratories Inc., USA). The button was implanted subcutaneously as previously described^24^, enabling repeated intravenous access.

Treatment compounds or PBS control were administered in a total daily volume of 250 µL using sterile phosphate-buffered saline without calcium or magnesium (PBS^−/−^). RNase A (Sigma-Aldrich/Merck KGaA) was dosed at 0.1 µg/g once daily. DNase I (Sigma-Aldrich/Merck KGaA) was administered at 7.5 µg/g body weight, twice daily. Dosages were selected based on previously published protocols^16,27^.

At the experimental end point, mice were euthanized with a ketamine/xylazine overdose. Animals were then perfused with PBS^−/−^ followed by 4% paraformaldehyde and aortic aneurysms were carefully dissected and processed for histological analysis and RNA isolation. Whole blood was collected for hematological profiling and for plasma processing. Complete blood count (CBC) analysis was performed using the Vet abc Hematology System (Lab Technologies, Austria).

### Histology

Collected aortas were fixed overnight in 4% paraformaldehyde, then processed and embedded in paraffin. Serial cross-sections (3 μm) were prepared for immunofluorescent and histological staining. Sections were deparaffinized in xylene and rehydrated through a graded ethanol series.

For immunofluorescence, antigen retrieval was performed using 1x citrate buffer (Thermo Fisher Scientific, USA), and tissues were permeabilized in 0.1% Triton X-100 diluted in blocking buffer of 5% bovine serum albumin in PBS. Macrophages were stained using a primary rat anti-mouse CD68 antibody (clone FA-11, 1:50 dilution in blocking buffer; Bio-Rad Laboratories Inc., USA), followed by a donkey anti-rat DyLight 650–conjugated secondary antibody (1:200 dilution in blocking buffer; Invitrogen, Thermo Fisher Scientific). Smooth muscle cells were identified using a Cy3-conjugated mouse anti-mouse α-smooth muscle actin (SMA) antibody (clone 1A4, 1:500 dilution in blocking buffer; Merck KGaA, Germany). Nuclei were counterstained with Hoechst 33342 (Thermo Fisher Scientific). Consecutive tissue sections were stained with Masson’s trichrome following the manufacturer’s protocol (Polysciences, USA).

Images were acquired and processed using an automated Axio Observer Z1 microscope (Carl Zeiss Microscopy GmbH, Germany) equipped with a 20x objective and the scanning software TissueFAXS Viewer (V7.1, TissueGnostics GmbH, Austria; https://tissuegnostics.com/products/scanning-and-viewing-software/tissuefaxs-imaging-software). Immunofluorescence staining was semi-quantitatively assessed by using a 4-point ordinal scale (0 = no signal, 1 = few positive cells, 2 = moderate, 3 = extensive coverage) as previously described^28^.

### Plasma exRNA isolation and quantification

exRNA was isolated from plasma samples using the Monarch Total RNA Miniprep Kit (New England Biolabs, USA), according to the manufacturer’s protocol for RNA purification from mammalian whole blood, including an on-column DNase I digestion step. For murine samples, 50 µL of EDTA plasma was processed per extraction, while 200 µL of citrate-theophylline-adenosine-dipyridamole plasma was used for human samples.

Following isolation, exRNA was quantified using the Quantus Fluorometer (Promega Corporation, USA) according to the manufacturer’s instructions for measuring RNA concentration using the supplied RNA standard to generate a standard curve. The total RNA amount measured in the eluted volume (30 µL) was used to calculate the original concentration of exRNA in plasma. Specifically, the total RNA (in nanograms) was divided by the starting plasma volume (50 µL for mouse samples, 200 µL for human samples), yielding the final exRNA concentration reported as ng/mL of plasma.

### Plasma exDNA isolation and quantification

exDNA was isolated from 50 µL of EDTA-treated mouse plasma (diluted with 150 µl PBS^−/−^) or 190 µL citrated human plasma using the DNeasy Blood & Tissue Kit (Qiagen, Germany), following the manufacturer’s protocol for purification of total DNA from animal blood or cells. DNA was eluted in 30 µL of nuclease-free water or AE buffer (component of DNeasy kit).

For sensitive detection of mouse exDNA, quantitative PCR (qPCR) was performed using 2 µL of the eluted DNA per reaction on a 7500 Fast Real-Time PCR System (Thermo Fisher Scientific), with SYBR Select Master Mix (Thermo Fisher Scientific). A designed primer pair targeting a 91 bp fragment of the mouse B1 repetitive element, analogous to human ALU sequences^29^, was used (forward: CGCCTTTAATCCCAGCACT; reverse: TGGCTGTCCTGGAACTCACT; 200nM). Relative quantification was calculated based on the amplification efficiency (*E*) of the primer set using the formula: ${E^{\left( {Calibrator~Cq - Sample~Cq} \right)}}$, where efficiency was determined from a serial dilution of mouse plasma DNA. A calibrator consisting of 100 pg of mouse spleen DNA was used as the reference sample for all reactions.

For human plasma samples, qPCR analysis of exDNA using ALU-based primers and quantification of citrullinated histone H3 (CitH3) by ELISA have previously been described^14^, and a subset of those samples is presented here for comparison.

### Aortic tissue RNA isolation, cDNA synthesis, and quantitative PCR

Total RNA was isolated from formalin-perfused abdominal aortic tissue using the Monarch Total RNA Miniprep Kit and recommended protocol for RNA purification from tissues (New England Biolabs). Frozen aneurysm samples were stored at – 80 °C until processing and homogenized in 300 µL of 1x DNA/RNA Protection Reagent (kit component) using CK14 tubes on a Precellys 24 homogenizer equipped with a Cryolys cooling module (Bertin Technologies, France). Following homogenization and proteinase K digestion, samples underwent an additional 15 min incubation at 65 °C to reverse formalin-induced crosslinking. On-column DNase I treatment was performed as recommended by the manufacturer. RNA was eluted in 30 µL of nuclease-free water and quantified using the NP80 Nanophotometer (Implen, Germany). The RNA integrity number (RIN) was assessed using the Agilent RNA 6000 Pico Kit and Agilent 2100 Bioanalyzer (Agilent Technologies, USA).

For each sample, 100–160 ng of RNA was reverse transcribed into complementary DNA (cDNA) using the High-Capacity cDNA Reverse Transcription Kit (Thermo Fisher Scientific) according to the manufacturer’s instructions.

qPCR was performed on a CFX Opus 96 Real-Time PCR System (Bio-Rad) using 3 ng of cDNA per reaction in a total volume of 20 µL. SYBR™ Select Master Mix (Thermo Fisher Scientific) was used with primer pairs (200 nM each) targeting *Mus musculus* HPRT1, TBP, MMP9, MYH11, and CNN1 (Supplementary Table 2). TaqMan™ Universal PCR Master Mix (Thermo Fisher Scientific) was used with commercial TaqMan™ Gene Expression Assays for *Mus musculus* ACTA2, MMP2, CCL2, GPX4, CD68, and MPO (Supplementary Table 2). Melting curve analysis was performed for SYBR-based reactions. Primer efficiencies were previously determined by standard curves for SYBR assays; TaqMan assay efficiency was assumed to be 2.0. Cq (cycle of quantitation) thresholds were set manually in CFX Maestro software (Bio-Rad) based on log-linear amplification regions and no-template-control signals. Cq values were averaged from technical duplicates; undetermined values in one replicate were omitted, while samples with both replicates undetermined were excluded. Relative quantities were calculated as ${E^{\left( {40 - Cq} \right)}},$ where *E* is the specific primer efficiency and 40 the theoretical maximum Cq (i.e. undetectable expression threshold). Relative quantities were then normalized to the geometric mean of the housekeeping genes, HPRT1 and TBP, expression. When gene expression measurements were performed on separate qPCR runs, five overlapping samples were included to allow for alignment with previous runs. All values were finally scaled to the sample with lowest transcript expression.

### Statistical analysis

Sample size calculations were based on our previously published treatment studies^18,28^. To detect a therapeutic reduction in AAA development with 80% statistical power and a significance level of 5% (α = 0.05), group sizes of 7 animals were determined to be sufficient. However, to account for model-specific conditions, such as an aneurysm rupture rate of ~ 35% prior to treatment start in the AngII model, as previously described by our lab^24^, additional animals were included in each experiment. As a result, the final group sizes shown in the plots represent the actual number of animals that developed an AAA prior to treatment initiation and survived until the experimental end point. Of note, only 1 mouse died of AAA rupture during drug therapy.

Data are presented as individual points with group means ± standard error of the mean (SEM). For AAA growth, measurements are expressed as percent change in aortic volume from baseline (100%). In treatment experiments, mice were stratified 1:1 with PBS-treated controls based on aortic volume at day 8 (AngII model) or day 4 (EPPE model), and comparisons between matched treatment-control pairs were performed using the Wilcoxon signed-rank test with significant differences indicated on the plots.

All other comparisons between groups were conducted using the Mann–Whitney U test. Spearman rank correlation was used to assess associations between continuous variables. Log₂ transformation (expression + 1) was applied to qPCR data prior to visualization and statistical testing.

All statistical analyses were performed using GraphPad Prism version 9.0.0 for Windows (GraphPad Software, USA), SPSS Statistics version 27.0 (IBM Corporation, USA), or Python version 3.11.4 (Python Software Foundation, USA). A p value < 0.05 was considered statistically significant.

## Results

### Plasma levels of exDNA but not exRNA increase during AAA development in mice and are elevated in AAA patients

**Fig. 1:**
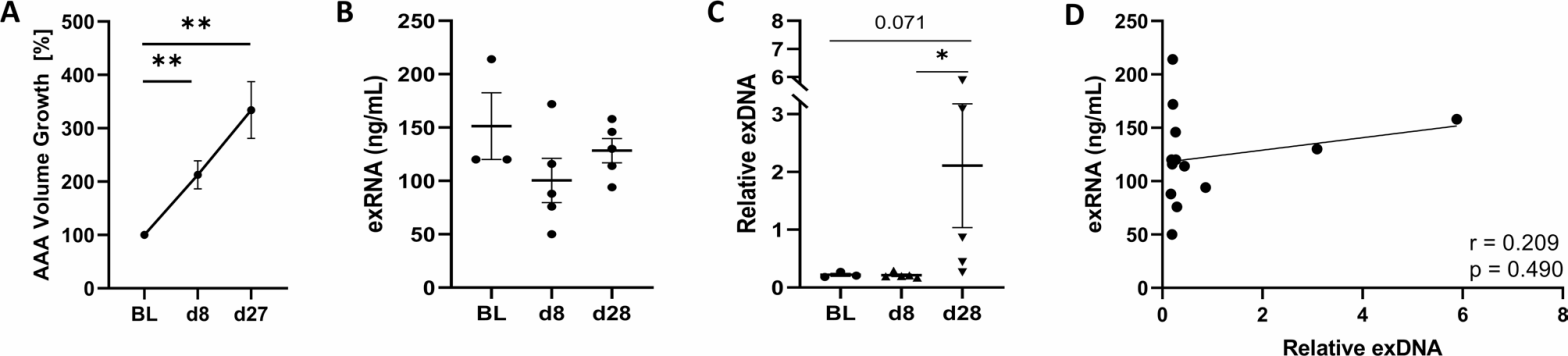
Circulating exRNA and exDNA in the AngII AAA mouse model. **A** Abdominal aortic aneurysm (AAA) volume changes over time in AngII-infused ApoE⁻^/^⁻ mice were determined by 3D ultrasound and expressed as percent change from baseline (BL, 100%) to day 8 and day 27. **B** Plasma concentrations of extracellular RNA (exRNA) were determined by fluorometric quantification and **C** relative plasma concentrations of extracellular DNA (exDNA) were measured by qPCR at these time points. **D** Spearman correlation analysis between exRNA and exDNA plasma levels from all time points was performed for AngII-treated mice. Values are presented as individual data points (*n* = 5 per group) with mean ± SEM (Mann–Whitney U test, ∗*p* < 0.05, ∗∗*p* < 0.01).

To evaluate whether circulating exRNA and exDNA reflect the release of extracellular nucleic acids during AAA development, their plasma concentrations were evaluated in preclinical models and in human subjects with AAA. In a mouse model of AAA induction by angiotensin II administration to ApoE deficient mice (AngII model), plasma samples were collected at baseline, day 8, and day 28. In this previously published time course of aneurysm development^28^, AAA volume (Fig. 1A) increased significantly from baseline (100%) to a mean value of 213% ± 26.3% SEM by day 8 (*p* = 0.004), and further to 334% ± 53.1% by day 27 (*p* = 0.004). exRNA levels (Fig. 1B) were measured via fluorometric analysis of RNA isolated from mouse plasma collected prior to AAA induction (baseline), at day 8 and at the experimental end point on day 28. Circulating exRNA showed no significant change over time (baseline: 151 ± 31.3 ng/mL vs. day 8: 100 ± 20.8 ng/mL vs. day 28: 128 ± 11.4 ng/mL). In contrast, exDNA (Fig. 1C) as quantified by qPCR, exhibited a significant increase in advanced disease from day 8 (0.213 ± 0.020) to day 28 (2.109 ± 1.071; *p* = 0.016), though no difference was observed in the early phase of AAA development from baseline (0.220 ± 0.026) to day 8. Of note, no correlation was found between exRNA and exDNA levels in mouse plasma (*r* = 0.209; Fig. 1D).

For AAA patients and human controls (*n* = 39 per group) matched in age, sex, body mass index, and smoking status, we previously reported that exDNA derived from nuclear chromatin was significantly elevated in AAA plasma compared to controls^14^. These data are consistent with our findings in the murine AngII model. For the current analysis, a subgroup of 10 AAA patients with high circulating exDNA (1.60 ± 0.234) and 10 controls with low exDNA levels (0.213 ± 0.014, *p* < 0.001; Supplementary Fig. 1A) were selected to assess whether exRNA levels similarly associate with disease status (demographics summarized in Supplementary Table 1). Yet, exRNA concentrations were only modestly higher in the AAA samples compared to controls (24.8 ± 9.28 ng/mL vs. 15.1 ± 3.13 ng/mL, statistically not significant; Supplementary Fig. 1B) and no correlation was observed between exRNA and exDNA levels in human plasma samples (*r* = -0.005; Supplementary Fig. 1C). Since the origin of circulating exDNA is thought to be primarily from neutrophil extracellular traps^5^, and we have previously reported a strong correlation between plasma exDNA and NET-derived citrullinated histone 3 (CitH3) levels in human AAA^14^, we next assessed this relationship in our selected human samples. In line, plasma CitH3 levels were strongly correlated with exDNA (*r* = 0.859; Supplementary Fig. 1D), whereas the association between CitH3 and exRNA was weak (*r* = 0.144; Supplementary Fig. 1E).

To determine whether the elevated circulating exDNA levels detected in patients and mice with AAA were consistent across murine AAA models, we further analyzed plasma exDNA in the external porcine pancreatic elastase (EPPE) model over time (Supplementary Fig. 2A) where AAA volume significantly grew to 196 ± 24.1% by day 4 (*p* = 0.008) and to 390 ± 75.3% by the end point (*p* = 0.004) in comparison to baseline (100%). Plasma exDNA levels were measured at baseline (0.322 ± 0.022), and day 14 (0.707 ± 0.153) following elastase application and were significantly elevated at the experimental end point on day 14 in comparison to baseline (*p* = 0.036; Supplementary Fig. 2B), thus consistent with findings in the AngII model. In a larger cohort of mice from each model, plasma exDNA levels at the experimental end point (Supplementary Fig. 2C) showed a trend toward higher circulating exDNA in the AngII model compared to the EPPE model (1.83 ± 0.652 vs. 0.536 ± 0.078; *p* = 0.093).

### Extracellular RNA degradation attenuates AAA progression in established AAA disease

**Fig. 2:**
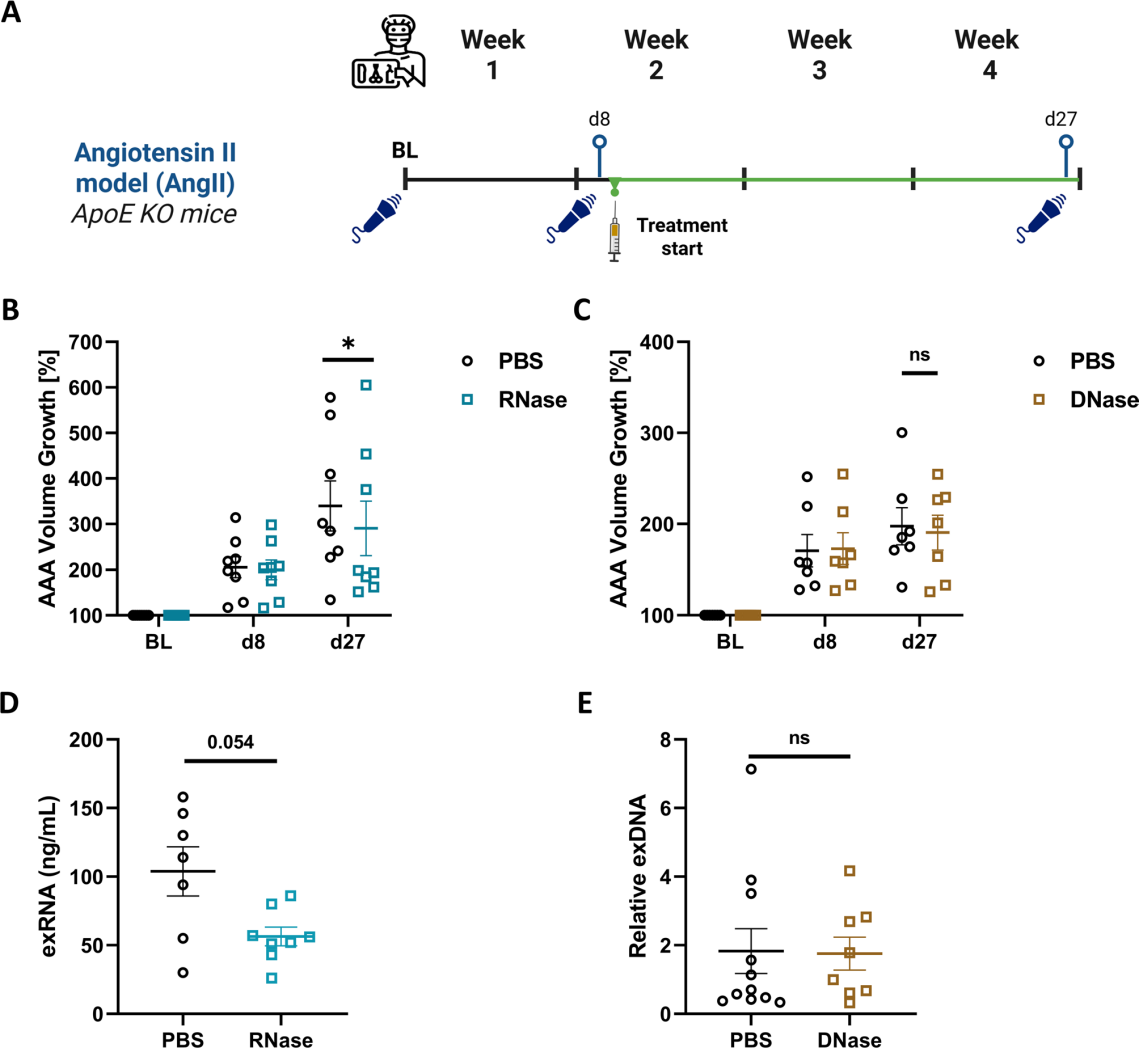
Treatment effect of extracellular nucleic acid degradation on established AAA in the AngII mouse model. **A** Schematic of the experimental design. ApoE⁻^/^⁻ mice underwent baseline ultrasound prior to continuous AngII infusion via Alzet osmotic pump. Aneurysm development was assessed at day 8, and mice were stratified into treatment groups based on 1:1 matching of aortic volume growth. Intravenous treatments (RNase A, DNase I, or PBS) were administered daily starting on day 9 via a jugular vein catheter and continued until day 27, when final aortic volume was measured. **B** Percent aortic volume growth from baseline (BL, 100%) for mice treated with RNase A and their matched PBS cohort (*n* = 8 per group). **C** Percent aortic volume growth from baseline (BL, 100%) for mice treated with DNase I or PBS (*n* = 7 per group). Blood was collected at the experimental end point (24 h after the last treatment). The plasma concentrations of **D** exRNA were determined by fluorometric quantification and **E** the relative plasma concentrations of exDNA were measured by quantitative PCR. Values are presented as individual points with mean ± SEM (Wilcoxon signed-rank test for paired comparisons; Mann–Whitney U test for circulating extracellular nucleic acid data; ∗*p* < 0.05, ns = not significant).

To assess the therapeutic impact of degrading extracellular nucleic acids on AAA progression in the AngII model, we allowed aneurysms to develop until day 8, then mice were stratified into treatment and control groups based on 1:1 matching of aortic volume growth (see experimental schematic in Fig. 2A). RNase A (to degrade exRNA), DNase I (to degrade exDNA), or PBS (control) were then administered intravenously daily starting at day 9 via an externalized vascular access button connected to the jugular vein. Treatment continued until day 27, at which point the final aortic volume was assessed by 3D ultrasound. The DNase I treatment group and its matched PBS control group were previously published and are shown here for comparison^28^.

In the RNase A experiment, treatment and control mice showed comparable aneurysm growth prior to treatment administration at day 8 (200 ± 22% vs. 206 ± 23%; Fig. 2B). Mice treated with RNase A exhibited moderately but significantly attenuated AAA progression compared to PBS-treated controls (final aortic volume growth: 291 ± 59% vs. 340 ± 55%; *p* = 0.039). In contrast, DNase I treatment did not affect aneurysm growth (final aortic volume growth: 191 ± 19% vs. PBS 198 ± 20%, *p* = 0.81; Fig. 2C). At the experimental end point (i.e., 24 h after the last nuclease administration), RNase A treated mice showed a reduction in plasma exRNA levels (56 ± 6.8 ng/mL vs. 104 ± ng/mL in PBS; *p* = 0.054; Fig. 2D). In contrast, DNase I treatment was not reflected in lower circulating exDNA levels at this sampling point (1.8 ± 0.48 vs. 1.8 ± 0.65 in PBS; Fig. 2E).

To assess whether systemic nucleic acid degradation may also modulate aneurysm progression in the EPPE model, C57BL/6J mice were similarly treated daily with RNase A, DNase I, or PBS from day 5 to day 13 following aneurysm induction, as shown in the experimental schematic (Supplementary Fig. 2D). Aortic volume growth at the end of the experiment did not differ significantly between RNase A and PBS control group (306 ± 42% vs. 316 ± 20%, *n* = 9, *p* = 0.570; Supplementary Fig. 2E). Notably, the DNase group exhibited a modest reduction in average aortic volume compared to PBS (261 ± 24% vs. 316 ± 28%, *n* = 7; Supplementary Fig. 2F), as we have previously reported^28^; however, this did not reach statistical significance.

### RNase A treatment reduces macrophage infiltration and preserves vascular smooth muscle cells in the AngII model

**Fig. 3:**
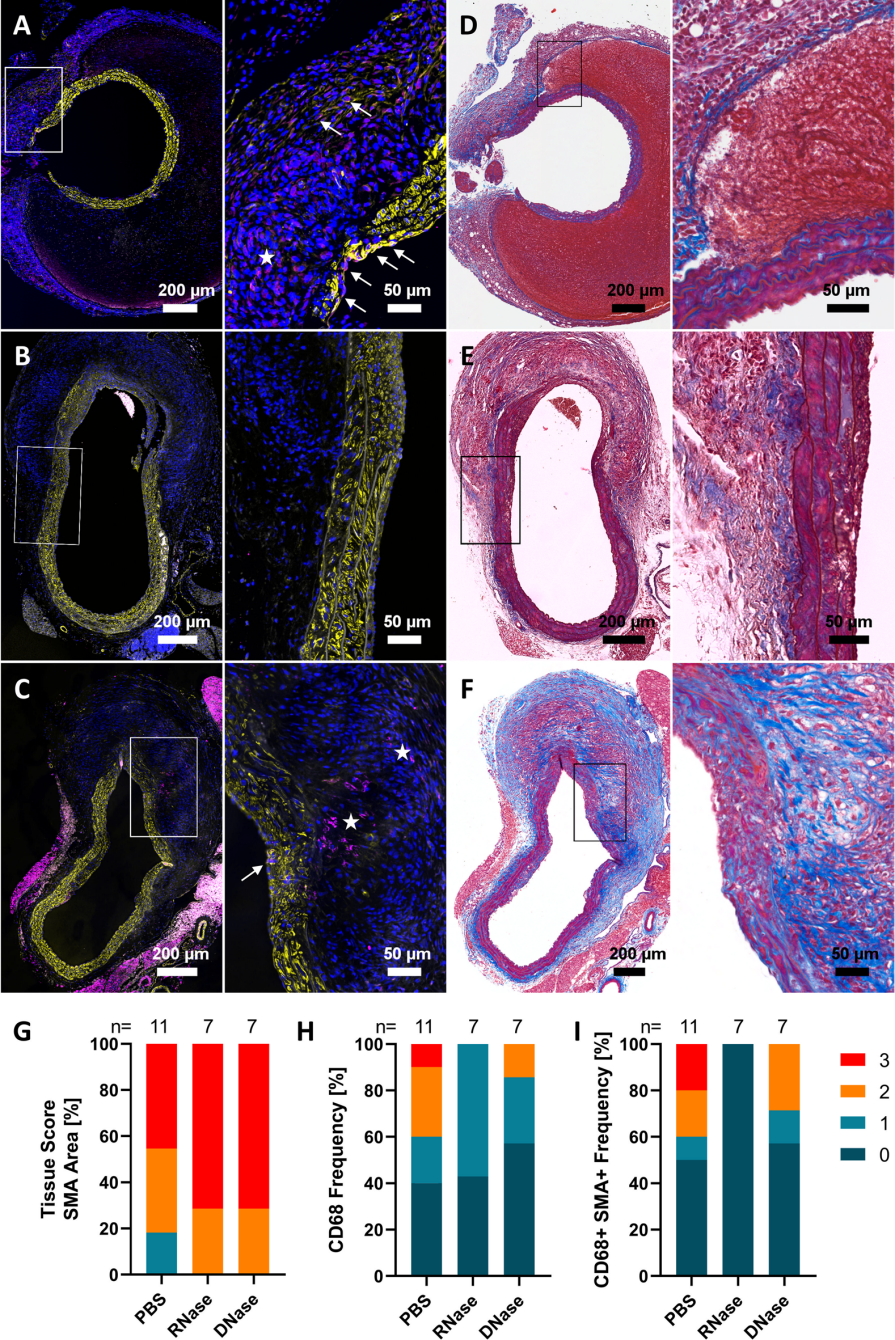
Alterations of macrophage infiltration and SMC phenotype in response to RNase A treatment in the AngII model. AAA tissue was harvested at the end point of experiments described in Fig. 2. Representative immunofluorescence images of aortic cross-sections from **A** PBS, **B** RNase or **C** DNase treated mice, stained for SMA (yellow), CD68 (magenta), and DNA (blue). Autofluorescence of elastin and collagen fibers is shown in gray. Masson’s trichrome staining of consecutive serial sections from **D** PBS, **E** RNase or **F** DNase treated mice. Marked areas of interest are shown as zoomed-in images. CD68^+^ areas of interest are indicated with a star, while CD68^+^SMA^+^ cells are marked with arrows. Semi-quantitative tissue scoring (0–3) was performed for **G** SMA⁺ area coverage, **H** CD68⁺ cell frequency, **I** CD68⁺ SMA⁺ co-localization, representing transdifferentiating smooth muscle cells (*n* = 11 for PBS group, *n* = 7 for RNase A and DNase I groups). Values are presented as stacked bars showing percent of mice per score. Of note, PBS and DNase groups were previously reported^28^ and are shown here for comparison; no statistical tests were applied.

To evaluate the impact of extracellular nucleic acid degradation on vascular remodeling and inflammation in AAA, aortic cross-sections from PBS-, RNase A-, and DNase I-treated AngII mice were stained for smooth muscle actin (SMA) and CD68 (Fig. 3A–C), or Masson’s trichrome (Fig. 3D–F), and manually scored 0–3 for area covered by SMA⁺ cells, frequency of CD68⁺ cell infiltration, and CD68⁺ SMA⁺ co-localization.

SMA staining revealed preserved smooth muscle coverage in both RNase- and DNase-treated mice (Fig. 3G). In the PBS group, 18% of mice had a low SMA signal indicating loss of contractile smooth muscle features, while in the treatment groups, SMA expression was preserved, in fact 71.4% of RNase- and DNase-treated mice showed extensive signal as compared to only 45.5% of PBS mice.

CD68 staining revealed a marked reduction in macrophage infiltration in RNase-treated mice (Fig. 3H). In the PBS group, 40% of aortas had no detectable CD68⁺ cells, while 60% exhibited moderate to high CD68 signal. In contrast, 100% of RNase-treated aortas showed low or undetectable CD68 staining, and DNase treatment also reduced CD68^+^ cell frequency, though some infiltration remained.

Double-positive CD68⁺ SMA⁺ signal, representing a subset of transdifferentiating SMCs, was entirely absent in the RNase group (100% of sections scored as negative), while 50% of PBS and 42.9% of DNase-treated mice showed cells of this phenotype (Fig. 3I). Additional elastin staining (data not shown) confirmed extensive degradation across all groups, with no pronounced differences between treatments.

### Aortic gene expression changes in the AngII model are mostly transient, showing a significant reduction of MMP9 by RNase A treatment at the experimental end point

Since RNase A was more effective than DNase I in reducing AAA growth in the AngII model, we aimed to further characterize the impact of RNase A treatment at the tissue gene expression level. To evaluate transcriptional changes associated with extracellular RNA degradation, qPCR was performed on abdominal aortic tissue harvested at the experimental end point. Genes were categorized into SMC markers, granulocyte lineage-related genes, and monocyte lineage-related genes.

The markers of interest were first evaluated in a time course experiment of AngII-treated mice (Supplementary Fig. 3), where multiple genes showed transient regulation at day 8 but expression levels had returned to baseline by day 28. Among SMC markers, SMA/ACTA2 transcripts increased at day 8 (3.14 ± 0.25) relative to baseline (2.17 ± 0.24; *p* = 0.057), followed by a significant decline by day 28 (1.74 ± 0.17; *p* = 0.016 vs. d8). Two other markers of the contractile SMC phenotype, myosin heavy chain 11 (MYH11) and calponin 1 (CNN1), showed a similar pattern of mRNA expression. Regarding granulocyte-related genes, myeloperoxidase (MPO) increased at day 8 (6.22 ± 1.24) vs. baseline (4.61 ± 1.56) and remained elevated at day 28 (6.57 ± 1.97). In contrast, the expression of glutathione peroxidase 4 (GPX4), known to protect against oxidative damage and ferroptosis, declined progressively with disease (3.14 ± 0.59 at baseline, 2.27 ± 0.18 at day 8, and 1.80 ± 0.28 at day 28). The neutrophil- (and also monocyte-) secreted matrix metalloproteinase MMP9 was only slightly elevated at day 8 (2.48 ± 0.32 vs. 2.17 ± 0.15) but increased markedly by day 28 (4.12 ± 0.89). Among monocyte-associated genes, monocyte chemoattractant protein-1 (MCP-1, also known as CCL2) was significantly upregulated at day 8 (3.86 ± 0.47) compared to baseline (1.77 ± 0.13; *p* = 0.029), and declined partially by day 28 (2.89 ± 0.88). The monocyte/macrophage markers CD68 and MMP2 followed a similar gene expression pattern.

**Fig. 4:**
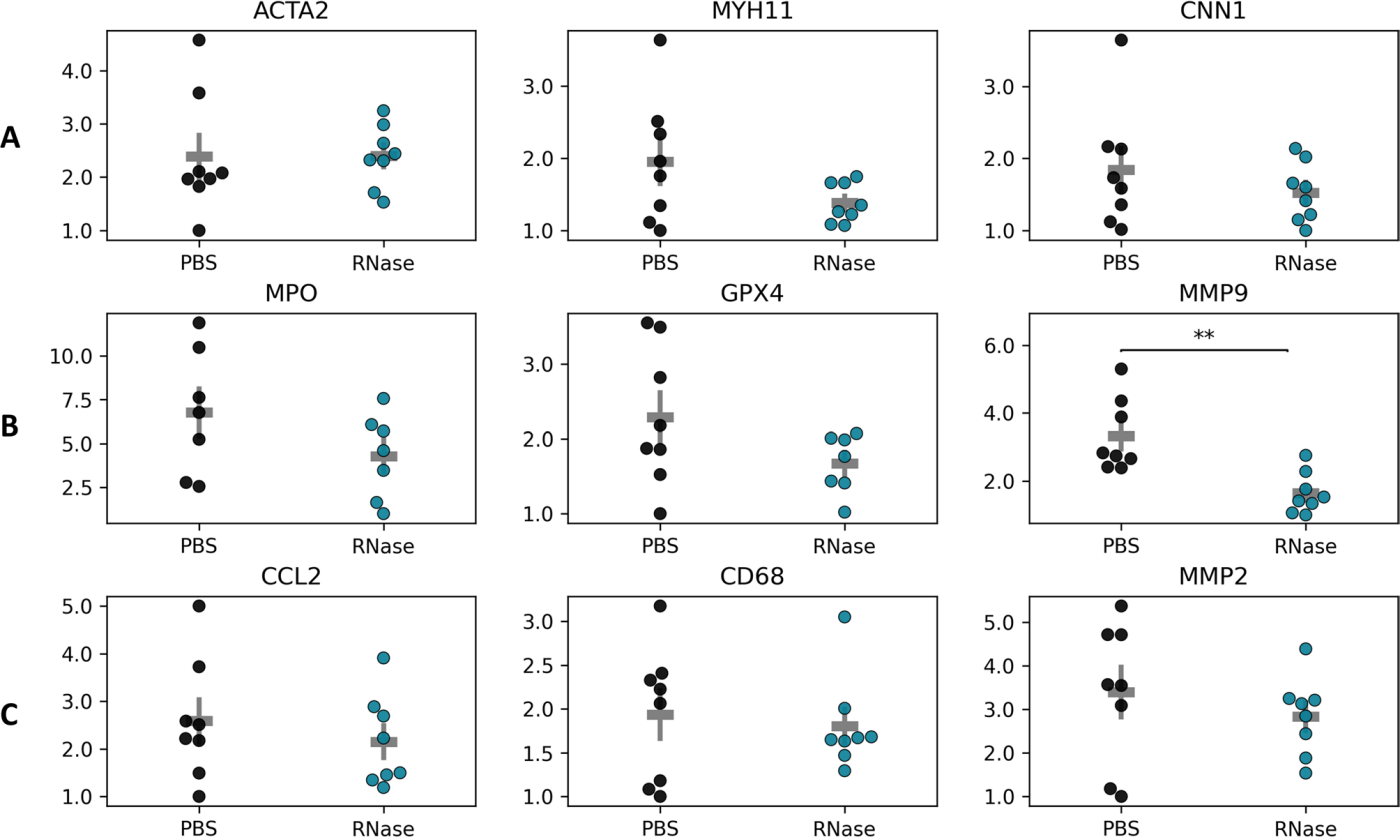
Effects of RNase A treatment on aortic gene expression in the AngII AAA model. Abdominal aortic tissues from PBS- and RNase A-treated mice (*n* = 8 per group) were harvested at the experimental end point on day 28 for RNA extraction, reverse transcription, and quantitative PCR analysis. Genes were grouped into **A** SMC markers, **B** granulocyte lineage–associated genes, and **C** monocyte lineage–associated genes. Values are presented as individual log transformed data points with group means ± SE indicated by gray lines. Statistical comparisons between groups were performed using the Mann–Whitney U test. Gene names, synonyms, and primer details are provided in Supplementary Table 2.

These genes of interest were then compared in AAA tissue of RNase A-treated mice and a matched PBS control cohort (*n* = 8 per group) at the experimental end point (day 28; Fig. 4). The expression of MMP9 was significantly reduced in the RNase A cohort (1.64 ± 0.21) in comparison to PBS controls (3.32 ± 0.38; *p* = 0.002) indicating a local decrease in proteolytic enzymes. Additionally, MPO showed a trend for lower expression in RNase A (4.29 ± 0.91) versus PBS-treated animals (6.76 ± 1.35) pointing to a reduction in oxidative stress, although this difference did not reach statistical significance. For SMC markers (SMA/ACTA2, MYH11, CNN1) and monocyte-related genes (CCL2, CD68, MMP2) whose transcript levels tended to return to baseline values by day 28 in the time course experiment, no significant differences were observed between RNase A- and PBS-treated mice at the experimental end point.

Of note, we previously also reported the transcript profile of AAA tissue from DNase I treated-mice^28^ which—consistent with the lack of therapeutic efficacy—did not reveal a gene expression pattern indicating reduced proteolysis or inflammation.

### RNase A treatment alters circulating leukocyte composition and reduces platelet counts in the AngII model

**Fig. 5:**
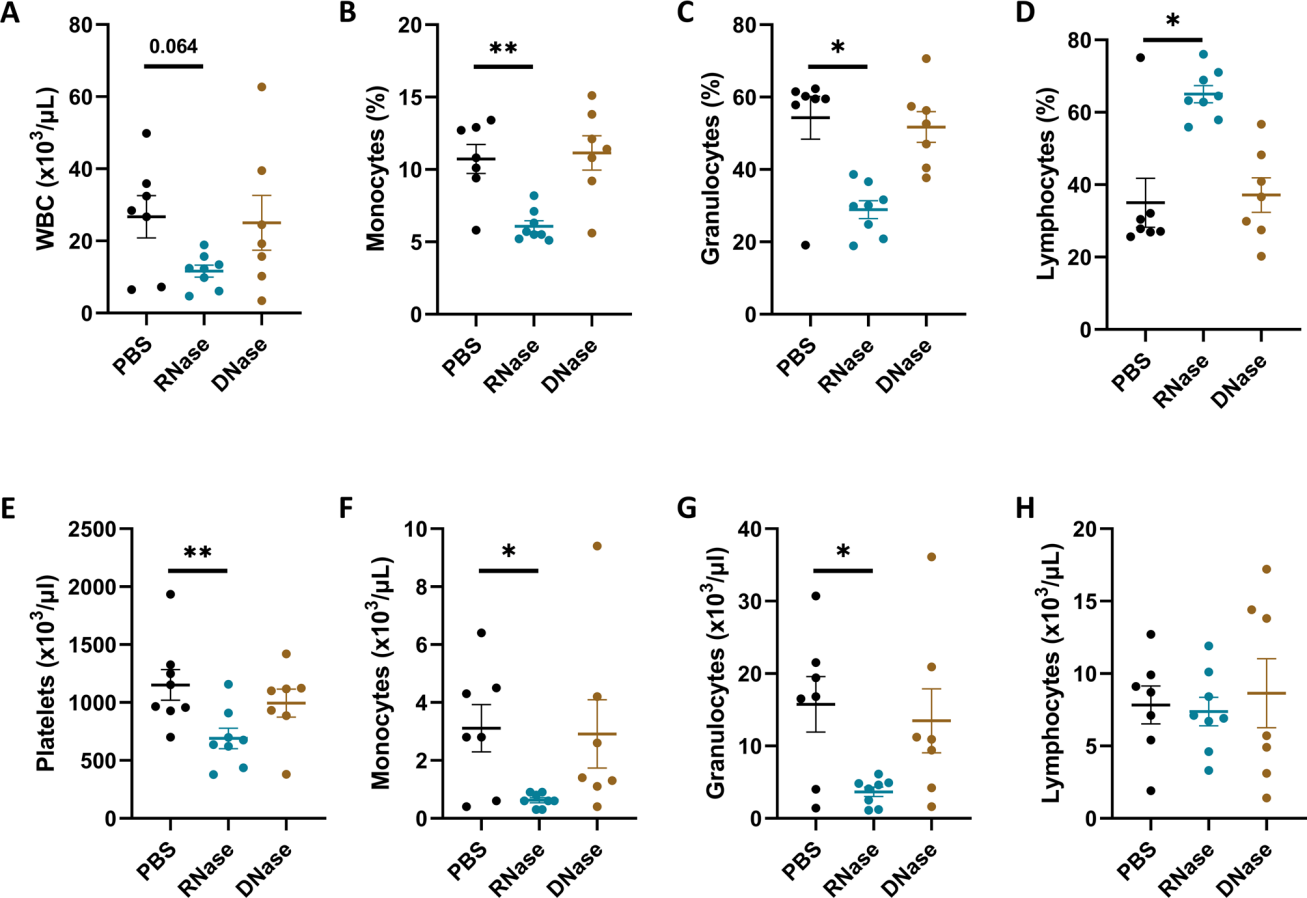
Nuclease-triggered changes of circulating leukocyte and platelet counts in the AngII-induced AAA mouse model. Whole blood was collected from PBS-, RNase A-, and DNase I-treated AngII mice at the experimental end point and analyzed using an automated hematology analyzer. **A** Total white blood cell (WBC) count, **B**–**D** percentages of monocytes, granulocytes, and lymphocytes, **E** platelet concentration, and **F**–**H** absolute counts of monocytes, granulocytes, and lymphocytes are shown. Values are presented as individual points with mean ± SEM. P values were calculated using Mann–Whitney U test (∗*p* < 0.05, ∗∗*p* < 0.01).

Since nucleases were administered intravenously, the local changes at the AAA site might in part be influenced by systemic treatment effects. Hence, we also assessed hematological responses to nuclease administration, as reflected in complete blood counts from RNase A-, DNase I-, and PBS-treated AngII mice at the experimental end point (Fig. 5). When comparing blood counts of PBS mice before and after AAA formation, we observed a significant increase in circulating monocytes and granulocytes at day 28 (Supplementary Fig. 4). Regarding treatment effects, the RNase group demonstrated a reduction to nearly baseline levels: total leukocyte counts were lower in the RNase group compared to PBS (11.66 ± 1.67 vs. 26.70 ± 5.86 × 10³/µL; *p* = 0.064). This difference was significantly driven by decreased monocyte (6.08 ± 0.38% vs. 10.7 ± 1.00%; *p* = 0.004) and granulocyte percentages (28.9 ± 2.48% vs. 54.3 ± 5.89%; *p* = 0.015), and these shifts were mirrored in absolute monocyte (0.63 ± 0.08 vs. 3.11 ± 0.82 × 10³/µL; *p* = 0.040) and granulocyte counts (3.66 ± 0.65 vs. 15.76 ± 3.83 × 10³/µL; *p* = 0.049). Lymphocyte counts remained unchanged (RNase: 7.38 ± 0.98 vs. PBS: 7.83 ± 1.31 × 10³/µL; *p* = 0.602), indicating that the increased lymphocyte percentage in RNase-treated mice reflects a relative redistribution due to the reduction of other leukocyte subsets rather than expansion of the lymphocyte pool.

In addition to leukocyte modulation, platelet counts were also significantly lower in RNase-treated mice (689.8 ± 88.2 vs. 1151.6 ± 132.2 × 10³/µL in PBS; *p* = 0.006).

No significant differences were observed in any measured blood parameters between DNase I- and PBS-treated mice.

## Discussion

In this study, we have addressed the role of extracellular nucleic acids in abdominal aortic aneurysm disease and identified exRNA rather than exDNA as a therapeutically targetable driver of AAA progression. Using systemic RNase A or DNase I administration in mice with established aneurysms, we found that RNase A treatment significantly attenuated further AAA growth in the angiotensin II triggered mouse model. In contrast, DNase I had no effect on aneurysm progression in established disease, which has been previously reported by us and others^16,28^. These findings highlight a specific functional role for exRNA in late-stage AAA progression.

Fischer et al. have shown that extracellular RNA but not DNA acts as a pro-inflammatory factor by promoting leukocyte recruitment in a murine model^21^. In line with this, RNase treatment was reported to reduce tissue infiltration by neutrophils and monocytes/macrophages in ischemic muscle disease^30^ and in atherosclerotic plaques of ApoE^−/−^ mice^31^. Our histological stainings of AAA tissue confirmed that RNase A treatment was accompanied by reduced CD68⁺ macrophage infiltration, and neutrophil markers such as MPO tended to be decreased in gene expression analysis of RNase treated mouse aneurysms. Strikingly, our study also revealed that RNase A but not DNase I induced a systemic decrease in circulating leukocytes, marked by significant reductions in monocytes and granulocytes in relative and absolute numbers. The systemic immune modulation aligned with effects at the aneurysm site, thus supporting a dual role for RNase A in dampening peripheral and local inflammatory responses during AAA progression.

At the tissue level, RNase A treatment was also associated with preservation of vascular smooth muscle cells. Histological scoring of SMA staining demonstrated improved coverage in RNase- and DNase-treated animals compared to PBS, but only RNase A treatment resulted in complete absence of CD68⁺SMA⁺ double-positive cells. This population has been proposed to represent transdifferentiating SMCs with macrophage-like features, a process increasingly recognized as a major contributor to vascular inflammation and matrix degradation^32^. Regarding transcript expression levels in AAA tissue of RNase A- and PBS-treated animals at the experimental end point, our analysis was limited by the circumstance that many of the investigated genes, including SMA/ACTA2, CCL2, CD68, and MMP2 exhibited peak expression changes on day 8 before treatment start but had largely returned to baseline by day 28 in control mice, leaving a small window of detection for therapeutic impact. Thus, the absence of significant gene expression differences at the end point likely reflects transcriptional normalization over time in the AngII model, rather than a lack of treatment effect. Yet, transcript levels of MMP9, a key matrix-degrading enzyme implicated in AAA development^33,34^, were significantly lower in RNase A- compared to PBS-treated mice, highlighting a potential mechanism by which RNase limits extracellular matrix breakdown. Since MMP9 is predominantly produced by activated myeloid cells^35^, this reduction in its expression aligns with the observed systemic decrease in circulating monocytes and granulocytes and the lower AAA macrophage infiltration following RNase A treatment.

Notably, MMP9 has been explored as a therapeutic target in AAA, including early-phase clinical studies using broad-spectrum MMP inhibitors such as doxycycline^36,37^, which have shown limited clinical efficacy. Given the multifactorial nature of AAA, the pleiotropic effects of RNase A and its low toxicity profile^38^ might offer a therapeutic advantage over single-target interventions. Additionally, since RNase A is administered intravenously, its therapeutic benefits likely comprise a combination of systemic and local effects within the aneurysm wall. By modulating several key pathological processes in parallel, RNase A may provide broader protection in a disease context where redundancy and compensatory mechanisms often undermine targeted approaches^1,2^. The lack of therapeutic response to RNase A in the second, elastase-induced mouse model may relate to the distinct trigger of AAA pathogenesis. While systemic AngII administration results in aneurysm formation with chronic inflammation and thrombus formation that shows substantial transcriptomic overlap with advanced human AAA disease, aneurysms of the EPPE model are induced by localized acute injury (without thrombus formation) representing disease initiation rather than progression^39,40^.

Interestingly, while targeting exRNA degradation by RNase A produced a significant therapeutic effect, circulating exRNA levels did not differ over time in the AngII-driven mouse model, nor between AAA patients and controls. This indicates that plasma levels of exRNA do not accurately reflect local concentrations within the aortic wall and are hence a poor biomarker of AAA disease. The fact that blood levels of exRNA showed no correlation with circulating CitH3, a marker of neutrophil extracellular trap formation, suggests a cellular origin distinct from NETs, possibly from necrotic or stressed non-myeloid cells or from extracellular vesicles^6^. In contrast, DNase I treatment had no impact on aneurysm growth but exDNA levels were consistently elevated in murine models and human AAA samples and strongly correlated with plasma CitH3. These findings may suggest that exDNA reflects NET-related inflammation in AAA, yet does not constitute a suitable therapeutic target, possibly due to compensatory inflammatory mechanisms^5^ or residual cytotoxic NET components^41,42^. As we have previously demonstrated, degradation of NET components is insufficient to curb AAA disease unless upstream signals are blocked to prevent NET formation^28^.

However, it is important to consider species differences: mice naturally have substantially higher basal DNase activity^43^ and a lower proportion of circulating neutrophils compared to humans^44^. Thus, the lack of efficacy with DNase I in established murine AAA may reflect an already compensated clearance system, whereas humans with lower endogenous DNase levels might still benefit from exogenous DNase therapy. Supporting this, DNase I was effective in a preclinical model when administered early after AAA induction during the acute inflammatory phase^17^ suggesting that therapeutic benefit depends on timing relative to inflammatory burden. Once AAA is established, endogenous DNase production in mice may suffice to control circulating exDNA, thereby limiting the additional therapeutic impact of DNase I supplementation that we have observed. In comparison, the plasma concentrations of RNase 1 are reported to be similar in mice and humans (~ 0.5 µg/mL)^45,46^, and extracellular RNA is stabilized in both species through conserved mechanisms such as vesicle packaging and RNA-binding proteins^47,48^. Although human RNase 1 has reportedly higher enzymatic activity and greater cellular uptake than its murine homolog^49,50^, there is no substantial evidence to date that the effects of RNase A observed in mice may not be translatable to human disease.

In conclusion, our findings identify extracellular RNA as a functionally relevant mediator of AAA progression. Systemic RNase A treatment can attenuate established AAA disease in a pre-clinical model, likely by modulating key pathological processes such as inflammation and extracellular matrix degradation. Unlike extracellular DNA, which could potentially serve as a biomarker rather than viable therapeutic target, exRNA has emerged as a targetable effector of advanced AAA disease, and systemic RNase treatment has potential as a novel, multi-modal treatment strategy in AAA—a condition for which no medical therapy currently exists.

Lastly, limitations of our study should be considered. While systemic concentrations of exRNA and exDNA were measured in plasma, their detection in AAA tissue was hampered by their low levels, fast degradation and formalin/paraffin treatment of samples. Similarly, the effect of nucleases on circulating exRNA and exDNA levels are difficult to ascertain at the experimental end point (i.e. 24 h after the last nuclease administration), since plasma levels of exRNA and exDNA are quickly restored and require daily nuclease administration. Indeed, RNase A administration showed a trend towards reduced circulating exRNA levels, while DNase I treatment was not reflected in lower exDNA levels at the end point. Regarding exRNA quantification, we performed RNA isolation and employed a fluorometric assay to detect total exRNA, which, while more sensitive than reported methods like NanoDrop or Bioanalyzer for low RNA concentrations, lacks the sensitivity of the qPCR used for exDNA. This might in part explain the better biomarker performance of circulating exDNA as compared to exRNA in AAA disease. The analyses were based on a limited set of samples, yet AAA patients and controls were carefully matched, differing only significantly in the AAA risk factor of hyperlipidemia and the previously reported AAA biomarker D-dimer^51^. According to ethics guidelines, group size in mouse experiments was minimized by the use of sensitive 3D ultrasound analysis of AAA growth^25^.

## Supplementary Information

Below is the link to the electronic supplementary material.

Supplementary Material 1

## Data Availability

The authors will make the raw data supporting the conclusions of this article available upon reasonable request to the corresponding author (Christine Brostjan).

## References

[1] Golledge, J., Thanigaimani, S., Powell, J. T. & Tsao, P. S. Pathogenesis and management of abdominal aortic aneurysm. *Eur. Heart J.* **44**, 2682–2697. 10.1093/eurheartj/ehad386 (2023). 37387260 10.1093/eurheartj/ehad386 PMC10393073

[2] Kessler, V., Klopf, J., Eilenberg, W., Neumayer, C. & Brostjan, C. AAA revisited: a comprehensive review of risk factors, management, and hallmarks of pathogenesis. *Biomedicines* **10**10.3390/biomedicines10010094 (2022). 10.3390/biomedicines10010094 PMC8773452 35052774

[3] Gao, J. P. & Guo, W. Mechanisms of abdominal aortic aneurysm progression: a review. *Vasc Med. (United Kingdom)*. **27**, 88–96. 10.1177/1358863X211021170 (2022). 10.1177/1358863X211021170 34278882

[4] Wanhainen, A. et al. (eds) ‘s Choice -- European Society for Vascular Surgery (ESVS) 2024 Clinical Practice Guidelines on the Management of Abdominal Aorto-Iliac Artery Aneurysms. *Eur. J. Vasc. Endovasc. Surg.* **67**, 192–331. 10.1016/j.ejvs.2023.11.002 (2024). 10.1016/j.ejvs.2023.11.002 38307694

[5] Artner, T., Sharma, S. & Lang, I. M. Nucleic acid liquid biopsies in cardiovascular disease: Cell-free DNA liquid biopsies in cardiovascular disease. *Atherosclerosis* **398**, 118583. 10.1016/j.atherosclerosis.2024.118583 (2024). 39353793 10.1016/j.atherosclerosis.2024.118583

[6] Sharma, S., Artner, T., Preissner, K. T. & Lang, I. M. Nucleic acid liquid biopsies in cardiovascular disease: cell-free RNA liquid biopsies in cardiovascular disease. *Atherosclerosis* **398**, 118584. 10.1016/j.atherosclerosis.2024.118584 (2024). 39306538 10.1016/j.atherosclerosis.2024.118584

[7] Polina, I. A., Ilatovskaya, D. V. & DeLeon-Pennell, K. Y. Cell free DNA as a diagnostic and prognostic marker for cardiovascular diseases. *Clin. Chim. Acta*. **503**, 145–150. 10.1016/j.cca.2020.01.013 (2020). 31978408 10.1016/j.cca.2020.01.013 PMC7042038

[8] Preissner, K. T. & Herwald, H. Extracellular nucleic acids in immunity and cardiovascular responses: between alert and disease. *Thromb. Haemost*. **117**, 1272–1282. 10.1160/TH-16-11-0858 (2017). 28594050 10.1160/TH-16-11-0858

[9] Fuchs, T. A. et al. Novel cell death program leads to neutrophil extracellular traps. *J. Cell. Biol.* **176**, 231–241. 10.1083/jcb.200606027 (2007). 17210947 10.1083/jcb.200606027 PMC2063942

[10] Brinkmann, V. et al. Neutrophil extracellular traps kill bacteria. *Science* **303**, 1532–1535. 10.1126/science.1092385 (2004). 15001782 10.1126/science.1092385

[11] Thorsen, S. U., Moseholm, K. F. & Clausen, F. B. Circulating cell-free DNA and its association with cardiovascular disease: what we know and future perspectives. *Curr. Opin. Lipidol.* **35**, 14–19. 10.1097/MOL.0000000000000907 (2024). 37800671 10.1097/MOL.0000000000000907

[12] Brusca, S. B. et al. Plasma cell-free DNA predicts survival and maps specific sources of injury in pulmonary arterial hypertension. *Circulation* **146**, 1033–1045. 10.1161/CIRCULATIONAHA.121.056719 (2022). 36004627 10.1161/CIRCULATIONAHA.121.056719 PMC9529801

[13] Jylhava, J. et al. Circulating cell-free DNA is associated with cardiometabolic risk factors: the health 2000 survey. *Atherosclerosis* **233**, 268–271. 10.1016/j.atherosclerosis.2013.12.022 (2014). 24529155 10.1016/j.atherosclerosis.2013.12.022

[14] Hayden, H. et al. Quantitation of oxidized nuclear and mitochondrial DNA in plasma samples of patients with abdominal aortic aneurysm. *Free Radic Biol. Med.* **206**, 94–105. 10.1016/j.freeradbiomed.2023.06.014 (2023). 37353175 10.1016/j.freeradbiomed.2023.06.014

[15] Delbosc, S. et al. Porphyromonas gingivalis participates in pathogenesis of human abdominal aortic aneurysm by neutrophil activation. Proof of concept in rats. *PLoS One*. **6**, e18679. 10.1371/journal.pone.0018679 (2011). 21533243 10.1371/journal.pone.0018679 PMC3076426

[16] Meher, A. K. et al. Novel role of IL (Interleukin)-1beta in neutrophil extracellular trap formation and abdominal aortic aneurysms. *Arterioscler. Thromb. Vasc. Biol.* **38**, 843–853. 10.1161/ATVBAHA.117.309897 (2018). 29472233 10.1161/ATVBAHA.117.309897 PMC5864548

[17] Yan, H. et al. Neutrophil proteases promote experimental abdominal aortic aneurysm via extracellular trap release and plasmacytoid dendritic cell activation. *Arterioscler. Thromb. Vasc. Biol.* **36**, 1660–1669. 10.1161/ATVBAHA.116.307786 (2016). 27283739 10.1161/ATVBAHA.116.307786 PMC4965335

[18] Eilenberg, W. et al. Histone citrullination as a novel biomarker and target to inhibit progression of abdominal aortic aneurysms. *Transl. Res.* **233**, 32–46. 10.1016/j.trsl.2021.02.003 (2021). 33571683 10.1016/j.trsl.2021.02.003

[19] Li, K. et al. Advances, challenges, and opportunities in extracellular RNA biology: insights from the NIH ExRNA strategic workshop. *JCI Insight*. **3**10.1172/jci.insight.98942 (2018). 10.1172/jci.insight.98942 PMC5928855 29618663

[20] Preissner, K. T., Fischer, S. & Deindl, E. Extracellular RNA as a versatile DAMP and alarm signal that influences leukocyte recruitment in inflammation and infection. *Front. Cell. Dev. Biol.* **8**, 619221. 10.3389/fcell.2020.619221 (2020). 33392206 10.3389/fcell.2020.619221 PMC7775424

[21] Fischer, S. et al. Extracellular RNA promotes leukocyte recruitment in the vascular system by mobilising Proinflammatory cytokines. *Thromb. Haemost.*. **108**, 730–741. 10.1160/TH12-03-0186 (2012). 22836360 10.1160/TH12-03-0186

[22] Lee, S. M. et al. Recognition of double-stranded RNA and regulation of interferon pathway by Toll-Like receptor 10. *Front. Immunol.* **9**, 516. 10.3389/fimmu.2018.00516 (2018). 29616030 10.3389/fimmu.2018.00516 PMC5865411

[23] Daugherty, A., Manning, M. W. & Cassis, L. A. Angiotensin II promotes atherosclerotic lesions and aneurysms in apolipoprotein E-deficient mice. *J. Clin. Investig.* **105**, 1605–1612. 10.1172/JCI7818 (2000). 10841519 10.1172/JCI7818 PMC300846

[24] Ibrahim, N. et al. Drug treatment by central venous catheter in a mouse model of angiotensin II induced abdominal aortic aneurysm and monitoring by 3D ultrasound. *J. Vis. Exp.*10.3791/64124 (2022). 35993760 10.3791/64124

[25] Ibrahim, N. et al. 3D ultrasound measurements are highly sensitive to monitor formation and progression of abdominal aortic aneurysms in mouse models. *Front. Cardiovasc. Med.* **9**, 944180. 10.3389/fcvm.2022.944180 (2022). 35903666 10.3389/fcvm.2022.944180 PMC9314770

[26] Bhamidipati, C. M. et al. Development of a novel murine model of aortic aneurysms using peri-adventitial elastase. *Surgery (United States)* **152**, 238–246. 10.1016/j.surg.2012.02.010 (2012). 10.1016/j.surg.2012.02.010 PMC3601193 22828146

[27] Stieger, P. et al. Targeting of extracellular RNA reduces edema formation and infarct size and improves survival after myocardial infarction in mice. *J. Am. Heart Assoc.* **6**10.1161/JAHA.116.004541 (2017). 10.1161/JAHA.116.004541 PMC5669142 28637776

[28] Ibrahim, N. et al. Reducing abdominal aortic aneurysm progression by blocking neutrophil extracellular traps depends on thrombus formation. *J. Am. Coll. Cardiol. Basic Trans. Sci.* **9**, 342–360. 10.1016/j.jacbts.2023.11.003 (2024). 10.1016/j.jacbts.2023.11.003 PMC10978405 38559632

[29] Quentin, Y. Successive waves of fixation of B1 variants in rodent lineage history. *J. Mol. Evol.* **28**, 299–305. 10.1007/BF02103425 (1989). 2471838 10.1007/BF02103425

[30] Lasch, M. et al. RNase A treatment interferes with leukocyte recruitment, neutrophil extracellular trap formation, and angiogenesis in ischemic muscle tissue. *Front. Physiol.* **11**, 576736. 10.3389/fphys.2020.576736 (2020). 33240100 10.3389/fphys.2020.576736 PMC7677187

[31] Simsekyilmaz, S. et al. Role of extracellular RNA in atherosclerotic plaque formation in mice. *Circulation* **129**, 598–606. 10.1161/CIRCULATIONAHA.113.002562 (2014). 24201302 10.1161/CIRCULATIONAHA.113.002562 PMC3946546

[32] Feil, S. et al. Transdifferentiation of vascular smooth muscle cells to macrophage-like cells during atherogenesis. *Circ. Res.* **115**, 662–667. 10.1161/CIRCRESAHA.115.304634 (2014). 25070003 10.1161/CIRCRESAHA.115.304634

[33] Freestone, T. et al. Inflammation and matrix metalloproteinases in the enlarging abdominal aortic aneurysm. *Arterioscler. Thromb. Vasc. Biol.* **15**, 1145–1151. 10.1161/01.atv.15.8.1145 (1995). 7627708 10.1161/01.atv.15.8.1145

[34] Takagi, H., Manabe, H., Kawai, N., Goto, S. N. & Umemoto, T. Circulating matrix metalloproteinase-9 concentrations and abdominal aortic aneurysm presence: a meta-analysis. *Interact. Cardiovasc. Thorac. Surg.* **9**, 437–440. 10.1510/icvts.2009.208835 (2009). 19525292 10.1510/icvts.2009.208835

[35] Parks, W. C., Wilson, C. L. & Lopez-Boado, Y. S. Matrix metalloproteinases as modulators of inflammation and innate immunity. *Nat. Rev. Immunol.* **4**, 617–629. 10.1038/nri1418 (2004). 15286728 10.1038/nri1418

[36] Meijer, C. A. et al. Doxycycline for stabilization of abdominal aortic aneurysms: a randomized trial. *Ann. Intern. Med.* **159**, 815–823. 10.7326/0003-4819-159-12-201312170-00007 (2013). 24490266 10.7326/0003-4819-159-12-201312170-00007

[37] Paghdar, S. et al. Doxycycline therapy for abdominal aortic aneurysm: inhibitory effect on matrix metalloproteinases. *Cureus* **13**, e14966. 10.7759/cureus.14966 (2021). 34123662 10.7759/cureus.14966 PMC8191685

[38] Kleinert, E. et al. Ribonuclease (RNase) prolongs survival of grafts in experimental heart transplantation. *J. Am. Heart Assoc.* **5**10.1161/JAHA.116.003429 (2016). 10.1161/JAHA.116.003429 PMC4889206 27121849

[39] Phillips, E. H. et al. Morphological and Biomechanical differences in the elastase and AngII ApoE -/- rodent models of abdominal aortic aneurysms. *BioMed. Res. Int.* **2015**10.1155/2015/413189 (2015). 10.1155/2015/413189 PMC4433642 26064906

[40] Gabel, G. et al. Parallel murine and human aortic wall genomics reveals metabolic reprogramming as key driver of abdominal aortic aneurysm progression. *J. Am. Heart Assoc.* **10**, e020231. 10.1161/JAHA.120.020231 (2021). 34420357 10.1161/JAHA.120.020231 PMC8649280

[41] Ibrahim, N., Eilenberg, W., Neumayer, C. & Brostjan, C. Neutrophil extracellular traps in cardiovascular and aortic disease: a narrative review on molecular mechanisms and therapeutic targeting. *Int. J. Mol. Sci.* **25**10.3390/ijms25073983 (2024). 10.3390/ijms25073983 PMC11012109 38612791

[42] Martinod, K. & Wagner, D. D. Thrombosis: tangled up in NETs. *Blood* **123**, 2768–2776. 10.1182/BLOOD-2013-10-463646 (2014). 24366358 10.1182/blood-2013-10-463646 PMC4007606

[43] Janovicova, L. et al. Sex, Age, and bodyweight as determinants of extracellular DNA in the plasma of mice: a cross-sectional study. *Int. J. Mol. Sci.* **20**10.3390/ijms20174163 (2019). 10.3390/ijms20174163 PMC6747214 31454899

[44] Mestas, J. & Hughes, C. C. Of mice and not men: differences between mouse and human immunology. *J. Immunol.* **172**, 2731–2738. 10.4049/jimmunol.172.5.2731 (2004). 14978070 10.4049/jimmunol.172.5.2731

[45] Weickmann, J. L., Olson, E. M. & Glitz, D. G. Immunological assay of pancreatic ribonuclease in serum as an indicator of pancreatic cancer. *Cancer Res.* **44**, 1682–1687 (1984). 6704974

[46] Garnett, E. R. et al. Phenotype of ribonuclease 1 deficiency in mice. *RNA* **25**, 921–934. 10.1261/rna.070433.119 (2019). 31053653 10.1261/rna.070433.119 PMC6633200

[47] Vickers, K. C., Palmisano, B. T., Shoucri, B. M., Shamburek, R. D. & Remaley, A. T. MicroRNAs are transported in plasma and delivered to recipient cells by high-density lipoproteins. *Nat. Cell. Biol.* **13**, 423–433. 10.1038/ncb2210 (2011). 21423178 10.1038/ncb2210 PMC3074610

[48] Valadi, H. et al. Exosome-mediated transfer of mRNAs and MicroRNAs is a novel mechanism of genetic exchange between cells. *Nat. Cell. Biol.* **9**, 654–659. 10.1038/ncb1596 (2007). 17486113 10.1038/ncb1596

[49] Eller, C. H., Lomax, J. E. & Raines, R. T. Bovine brain ribonuclease is the functional homolog of human ribonuclease 1. *J. Biol. Chem.* **289**, 25996–26006. 10.1074/jbc.M114.566166 (2014). 25078100 10.1074/jbc.M114.566166 PMC4176206

[50] Lomax, J. E., Eller, C. H. & Raines, R. T. Comparative functional analysis of ribonuclease 1 homologs: molecular insights into evolving vertebrate physiology. *Biochem. J.* **474**, 2219–2233. 10.1042/BCJ20170173 (2017). 28495858 10.1042/BCJ20170173 PMC5660862

[51] Golledge, J., Muller, R., Clancy, P., McCann, M. & Norman, P. E. Evaluation of the diagnostic and prognostic value of plasma D-dimer for abdominal aortic aneurysm. *Eur. Heart J.* **32**, 354–364. 10.1093/eurheartj/ehq171 (2011). 20530504 10.1093/eurheartj/ehq171

