# Supplementary material for: Extracellular RNA as a molecular driver and therapeutic target in abdominal aortic aneurysms
Source: Sci Rep. 2025 Oct 31;15:38266. doi: 10.1038/s41598-025-22041-y (PMC12578788; doi:10.1038/s41598-025-22041-y)
Supplement: Supplementary file 1 — Supplementary Material 1 [file 41598_2025_22041_MOESM1_ESM.pdf]

Supplementary Material

Extracellular RNA as a molecular driver and therapeutic target in abdominal aortic aneurysms

Nahla Ibrahim[1], Hubert Hayden[1], Gabriel Kurzreiter[1], Johannes Klopf[1], Sonja Bleichert[1], Tyler Artner[2], Alexander Stiglbauer-Tscholakoff[3], Wolf Eilenberg[1], Christoph Neumayer[1], Christine Brostjan[1*]

[1]Division of Vascular Surgery, Department of General Surgery, Medical University of Vienna and University Hospital Vienna, Vienna, Austria
[2]Division of Cardiology, Department of Internal Medicine II, Medical University of Vienna and University Hospital Vienna, Vienna, Austria
[3]Division of Cardiovascular and Interventional Radiology, Division of Molecular and Gender Imaging, Department of Biomedical Imaging and Image Guided Therapy, Medical University of Vienna and University Hospital Vienna, Vienna, Austria

*** Correspondence:**
Christine Brostjan
christine.brostjan@meduniwien.ac.at

List of Contents

1 Supplementary Tables 2
2 Supplementary Figures 4
3 Supplementary References 8

1 Supplementary Tables

Supplementary Table 1. Patient and control demographics.

**Characteristic**	**Control (n = 10)** *n (%)*	**AAA (n = 10)** *n (%)*	**p-value**
Sex			
Female	1 (10%)	1 (10%)	1.000
Male	9 (90%)	9 (90%)	
Smoker status			
Never	1 (10%)	0 (0%)	0.113*
Past	8 (80%)	5 (50%)	
Current	1 (10%)	5 (50%)	
Hypertension	4 (40%)	6 (60%)	0.371*
Hyperlipidemia	3 (30%)	9 (90%)	**0.020**
Peripheral artery disease	1 (10%)	3 (30%)	0.582
Coronary heart disease	0 (0%)	3 (30%)	0.211
Myocardial infarction	0 (0%)	2 (20%)	0.474
Stroke	1 (10%)	0 (0%)	1.000
Diabetes mellitus	0 (0%)	2 (20%)	0.474
COPD	0 (0%)	3 (30%)	0.211
Nephropathy/renal cysts	1 (10%)	3 (30%)	0.582
Carcinoma (current)	1 (10%)	0 (0%)	1.000
Autoimmune disease	1 (10%)	0 (0%)	1.000
	Median (IQR)	*Median (IQR)*	
Age [years]	63.5 (17.0)	67.1 (12.0)	0.545
Body mass index [kg/m^2]	25.0 (3.4)	27.4 (6.4)	0.821
White blood cells [G/L]	5.2 (2.7)#	6.3 (1.3)	0.165
Lymphocytes [G/L]	1.9 (0.8)#	1.4 (1.3)	0.967
Monocytes [G/L]	0.5 (0.3)#	0.5 (0.2)	0.616
Neutrophils [G/L]	3.2 (1.9)#	4.5 (1.3)	0.101
Red blood cells [T/L]	4.6 (1.1)#	4.5 (0.7)	0.566
Platelets [G/L]	241 (96)#	218 (98)	0.327
C-reactive protein [mg/dL]	0.16 (0.37)	0.22 (0.18)	0.762
D-dimer [µg/mL]	0.28 (0.66)#	1.44 (1.59)#	**0.011**
Maximum AAA diameter [mm]		61.1 (11.2)	
Aneurysm volume [cm^3]		136.4 (37.6)§	

AAA, abdominal aortic aneurysm; COPD, chronic obstructive pulmonary disease; IQR, interquartile range. P-values for categorical variables were calculated with Fisher´s exact test or Chi square test (*); p-values for metric variables were calculated with Mann-Whitney U test, and significant differences are labeled in bold; the lack of parameter values is indicated by # and § for 1 and 3 missing data entries, respectively.

Supplementary Table 2: Source of primer sequences applied in qPCR.

Gene ID	Gene/protein name	Detection method	Primer source
ACTA2	alpha-smooth muscle actin (SMA)	TaqMan™ Assay	Mm01546133_m1
CCL2	monocyte chemotactic protein 1 (MCP-1)	TaqMan™ Assay	Mm00441242_m1
CD68	macrosialin	TaqMan™ Assay	Mm03047343_m1
CNN1	calponin 1	SYBR Green™ Assay	[1]
GPX4	glutathione peroxidase 4	TaqMan™ Assay	Mm04411498_m1
HPRT1	hypoxanthine guanine phosphoribosyl transferase 1	SYBR Green™ Assay	[2]
MMP2	matrix metalloproteinase 2	TaqMan™ Assay	Mm00439498_m1
MMP9	matrix metalloproteinase 9	SYBR Green™ Assay	[3]
MPO	myeloperoxidase	TaqMan™ Assay	Mm01298424_m1
MYH11	myosin (heavy chain) 11	SYBR Green™ Assay	[4]
TBP	TATA box binding protein	SYBR Green™ Assay	[5]

2 Supplementary Figures

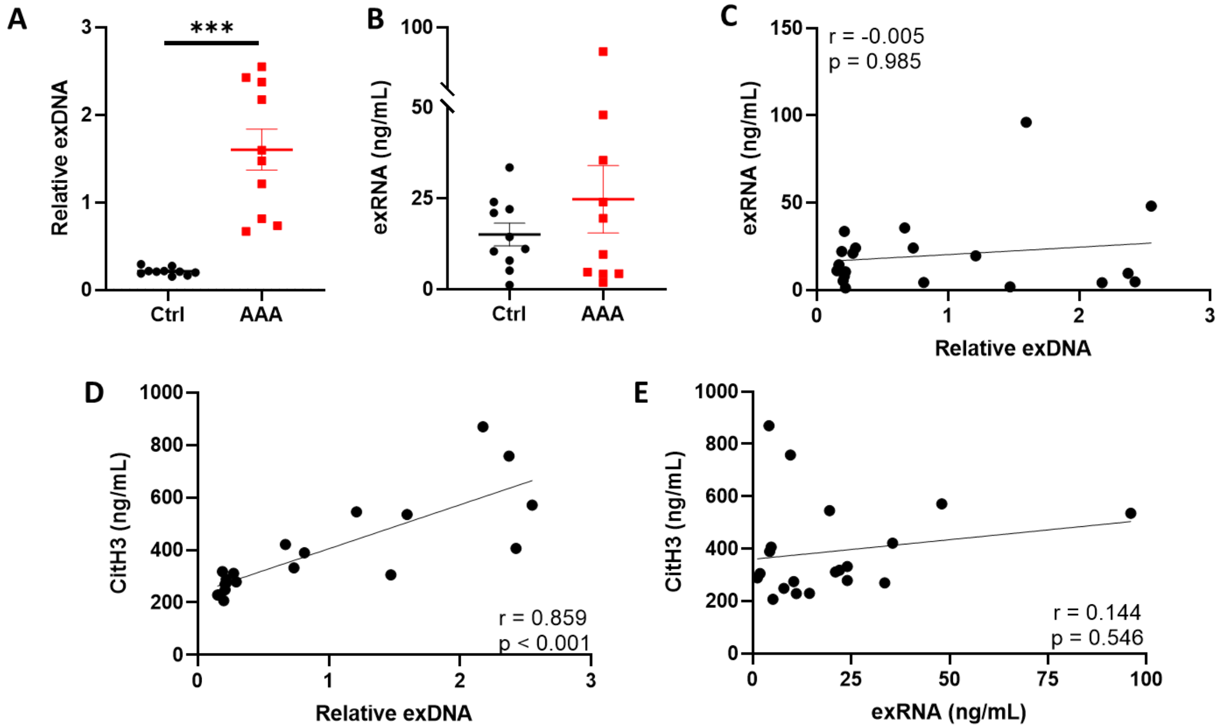

Supplementary Figure 1. Circulating levels of exDNA and exRNA and association with NET markers in human AAA disease. Plasma concentrations of (A) extracellular DNA (exDNA) and (B) extracellular RNA (exRNA) were measured in a subgroup of human AAA patients and matched controls (n = 10 per group) by qPCR and fluorometric quantification, respectively. Values are presented as individual points with mean ± SEM (Mann–Whitney U test, ∗∗∗p < 0.001). (C) Spearman correlation analysis between the circulating exRNA and exDNA levels. Correlation analysis between plasma levels of citrullinated histone 3 (CitH3) and (D) exDNA or (E) exRNA was performed for the selected human AAA samples (n = 20, AAA patients and controls, Spearman coefficient of correlation r).

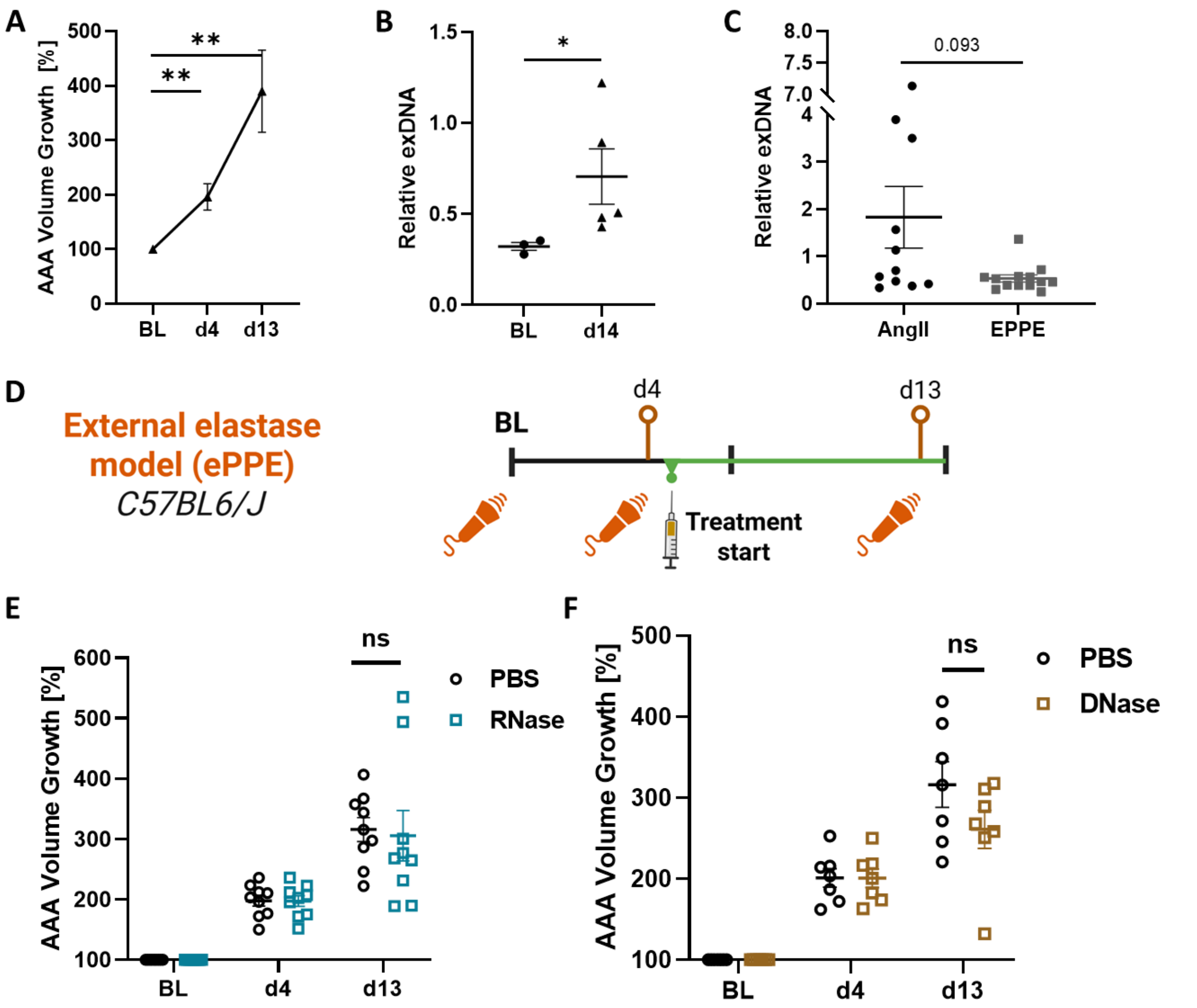

Supplementary Figure 2. Time course of AAA development and circulating exDNA levels in the EPPE mouse model and treatment effect of extracellular nucleic acid degradation. Aneurysms were induced by external porcine pancreatic elastase (EPPE) in C57BL/6J wild-type mice, (A) aneurysm growth was monitored by 3D ultrasound and AAA volume changes over time were expressed as percent of baseline (BL, 100%). (B) Plasma concentrations of exDNA were measured at BL and day 14 by qPCR and calculated relative to a set calibrator. (C) Plasma exDNA levels were compared at the experimental end point in a larger cohort of mice from the AngII and EPPE models (n = 11 vs 13). (D) Schematic of the therapeutic intervention design. Mice underwent baseline ultrasound analysis prior to aneurysm induction by peri-aortic elastase application. Aneurysm development was assessed at day 4, and mice were stratified into treatment groups based on 1:1 matching of aortic volume growth. Intravenous treatments (RNase A, DNase I, or PBS) were administered starting on day 5 via a jugular vein catheter and continued until day 13, when final aortic volume was measured. Percent aortic volume growth from baseline (100%) is shown for mice treated with (E) RNase A and the matched PBS cohort (n = 9 per group) and (F) DNase I and the matched PBS cohort (n = 7 per group). Values are presented as individual points with mean ± SEM. (Time course data was analyzed by Mann–Whitney U test, $*p < 0.05$, $**p < 0.01$; paired comparisons were analyzed by Wilcoxon signed-rank test, ns = not significant.)

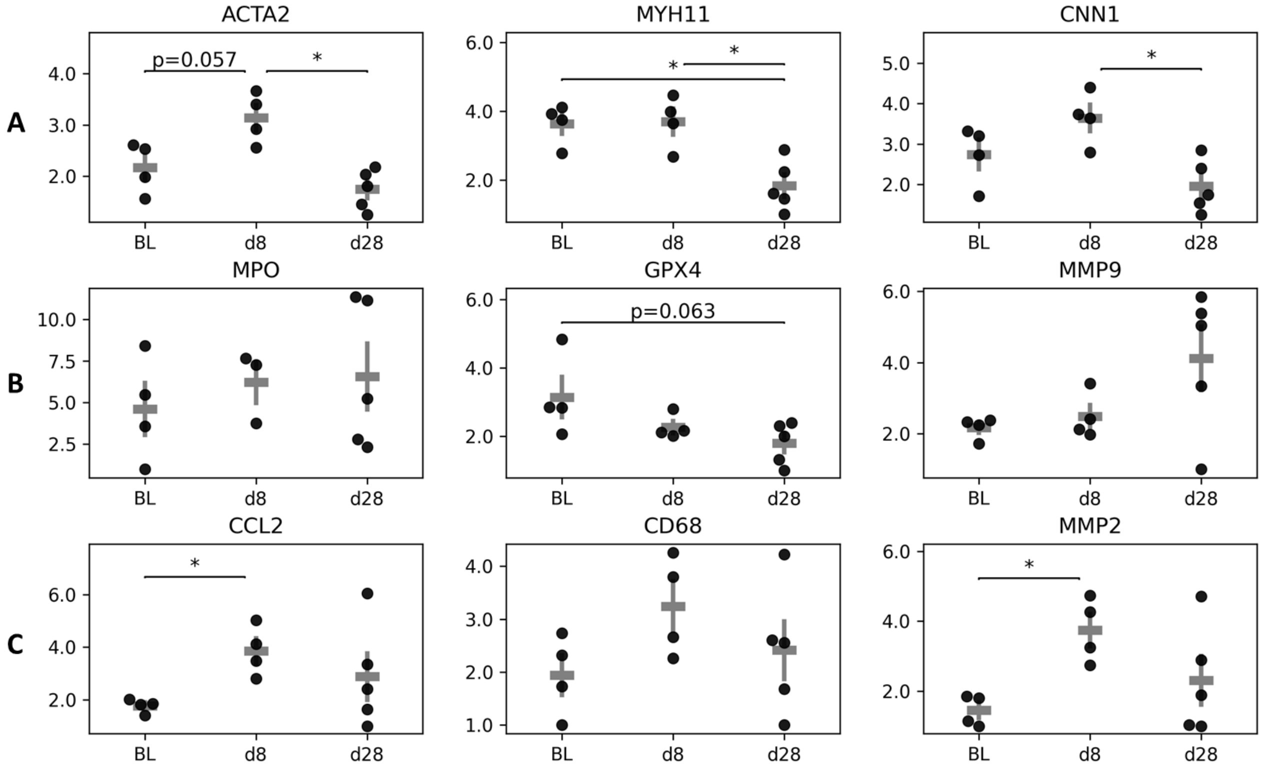

Supplementary Figure 3. Gene expression changes in aortic tissue during aneurysm development in the AngII AAA model. Quantitative PCR was performed on isolated RNA (cDNA) of abdominal aortic tissue collected from AngII-infused ApoE$^{-/-}$ mice at baseline (BL), day 8, and day 28 (PBS-treated). Different groups of mice (n = 4-5) were sacrificed at each time point to obtain tissue samples. Nine genes were selected to illustrate the temporal expression pattern of (A) key markers of SMC plasticity, (B) granulocyte lineage-associated genes, and (C) monocyte lineage-associated genes. Expression values are shown as individual log-transformed data points with group means ± SEM indicated by gray lines. Statistical comparisons between time points were performed using Mann–Whitney U test (*$p < 0.05$). Gene names, synonyms, and primer details are listed in **Supplementary Table 2**.

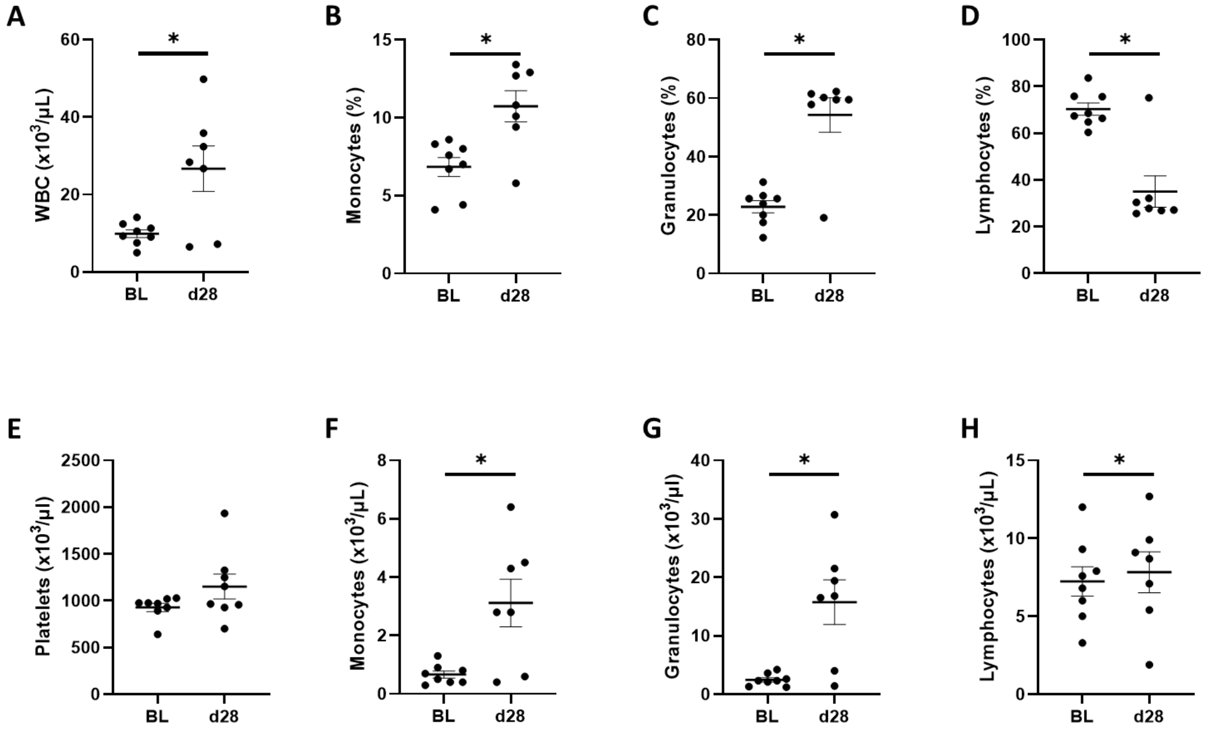

Supplementary Figure 4. Time course of AAA-induced changes in circulating leukocyte and platelet counts in the AngII mouse model. Whole blood was collected from PBS-treated AngII mice at baseline (BL: prior to AAA induction) and at the experimental end point (d28 post-AAA induction) and analyzed using an automated hematology analyzer. (A) Total white blood cell (WBC) count, (B–D) percentages of monocytes, granulocytes, and lymphocytes, (E) platelet concentration, and (F–H) absolute counts of monocytes, granulocytes, and lymphocytes are shown. Values are presented as individual points with mean ± SEM. P values were calculated using Wilcoxon signed-rank test ($*p < 0.05$). These baseline and end point measurements refer to the PBS-cohort shown in **Figure 5**.

3 Supplementary References

1. Li, J. *et al.* Young Bone Marrow Sca-1 Cells Rejuvenate the Aged Heart by Promoting Epithelial-to-Mesenchymal Transition. *Theranostics* **8**, 1766-1781 (2018). https://doi.org/10.7150/THNO.22788
2. Lee, D. D. & Schwarz, M. A. Adapted approach to profile genes while reconciling Vegf-a mRNA expression in the developing and injured lung. *American Journal of Physiology. Lung Cellular and Molecular Physiology* **308**, 1202-1211 (2015). https://doi.org/10.1152/AJPLUNG.00053.2015
3. Yen, J. H., Khayrullina, T. & Ganea, D. PGE2-induced metalloproteinase-9 is essential for dendritic cell migration. *Blood* **111**, 260-270 (2008). https://doi.org/10.1182/BLOOD-2007-05-090613
4. Long, X. *et al.* The smooth muscle cell-restricted KCNMB1 ion channel subunit is a direct transcriptional target of serum response factor and myocardin. *The Journal of Biological Chemistry* **284**, 33671-33682 (2009). https://doi.org/10.1074/JBC.M109.050419
5. Ho, K. H. & Patrizi, A. Assessment of common housekeeping genes as reference for gene expression studies using RT-qPCR in mouse choroid plexus. *Scientific Reports.* **11** (2021). https://doi.org/10.1038/S41598-021-82800-5
